# Microplastic Mass Estimation Using Two-Dimensional Chemical Images from Quantum-Cascade Laser-Based Infrared Spectrometers

**DOI:** 10.1021/acs.analchem.5c04003

**Published:** 2025-10-28

**Authors:** Borja Ferreiro, José M. Andrade, Adrián López-Rosales, Soledad Muniategui-Lorenzo

**Affiliations:** Grupo Química Analítica Aplicada (QANAP), Instituto Universitario de Medio Ambiente (IUMA), Centro de Innovación Tecnolóxica en Edificación e Enxeñería Civil (CITEEC), 16737 Universidade da Coruña, Campus da Zapateira, E-15071 A Coruña, Spain

## Abstract

Modern spectroscopic chemical imaging techniques perform nondestructive, relatively fast sample analysis to count and chemically characterize individual particles. However, they do not offer information on particle mass, a relevant parameter for several disciplines, like toxicology. This work strives to estimate the mass of plastic particles using a tiered sequence of steps and models that employ bidimensional parameters (height, width, perimeter, area) reported by state-of-the-art tunable quantum-cascade laser-based infrared imaging spectroscopy. A hybrid model that does not need calibration steps for its routine application is proposed for the first time. The shape of each particle is assessed individually, rather than setting them initially. Fibers are modeled as cylinders using an equivalent cylinder concept, while fragments are approximated to either parallelepipeds or spheroids, based on their 2D circularity. Previously published models were tested, modified, and hybridized to assess the mass of known sets of plastic particles whose size ranged from 20 to 1500 μm and whose total weight ranged from 190 to 9400 μg. The hybrid approach estimated the mass of exemplary samples with relative errors lower than 20% (a satisfactory level for these estimations) and worked well in size and weight ranges barely tested before. An Excel-based spreadsheet (NOMME, Number Of Microplastics and their Mass Estimation) was developed to streamline the application of the hybrid model.

## Graphical abstract

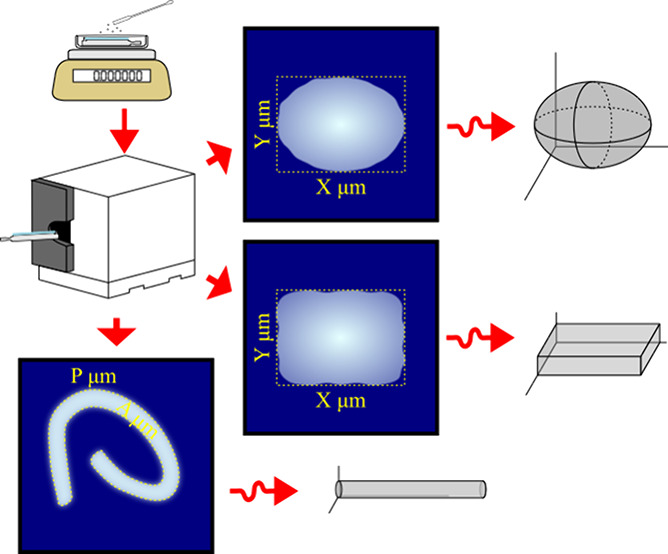

## Introduction

The immense quantity and widespread distribution of polymer residues worldwide attracted the attention of scientists, policymakers and the general public in the last years. Microplastic (MP) pollution has become increasingly relevant due to its ubiquity and potential harmful effects, whether direct (mostly physical affectation to biota) or/and indirect (due to toxic additives, adsorbed pollutants, etc.). MP studies pose significant scientific challenges, as they can be present in difficult-to-access areas, like deep-sea and oceanic trenches,^1^ Arctic and Antarctic areas^2,3^ or mountains.^4,5^ They can be either visible (like pellets in beaches) or be small and hidden inside organic and inorganic matrices (animal guts, sediments, vegetal tissues etc.). Their weathering may induce accelerated physical erosion and generate increasingly smaller particles which, along with biofouling and the adsorption of other contaminants, can alter the physicochemical composition of their surface.^6,7^ All these facts hinder their accurate characterization, as smaller particles are much more difficult to be isolated and measured, and the heavily weathered ones may not even yield identifiable analytical signals.

These difficulties have spurred technical innovation in order to stay in pace with the ever-increasing analytical demands of this field. Common spectroscopic techniques are hyphenated with microscopic or chemical imaging systems, like InfraRed (IR)^8,9^ and Raman^10^ spectroscopy. Pyrolysis-Gas Chromatography/Mass Spectrometry (Py-GC/MS)^11^ is also a powerful tool whose application is rising. Each analytical technique has its own advantages and disadvantages. Spectroscopic imaging offers rather fast results (thousands of particles per day), nondestructive analysis and the possibility to count and characterize individual particles both morphologically and chemically. On the other hand, chromatographic and mass spectrometric techniques offer low limits of mass detection, can measure the mass of the particles and sample preparation is usually simple; however, it is destructive and does not inform on individual particles.

To assess the toxicity of MPs it is important to gather as much information as possible from them: the polymer mass and number of particles in the samples give a sense of their general concentration in the matrix, while particle size affects the physical effects in the body. Nevertheless, evaluating the volume of the MPs and their mass is challenging because the only insights into their shape come from static 2D images (silhouettes or 2D projections of 3D particles) and, hence, it is highly difficult to derive accurately all relevant information.

A cutting-edge solution (accepted by ISO^12^ and European Union^13^) is to use chemical imaging instruments, like state-of-the-art tunable quantum-cascade laser-based Agilent’s LDIR imaging, to visualize, count, and identify the polymers. Afterward the volume and mass of the individual particles using descriptor parameters of their 2D images can be calculated, being this issue out of the scopes of the guidelines. To estimate the unknown volumes of the particles, several authors developed some approaches considering different geometric models. That was the case for Cózar et al.,^14^ who approximated the particles to cubes; Medina et al.^15^ that considered cylinders; Isobe et al.^16^ modeled them as spheres, while Simon et al.,^17^ Tanoiri et al.^18^ and Barchiesi et al.^19^ dealt with spheroids. The models, along with their geometrical basic parameters are illustrated in Figure 1a.

**Figure 1:**
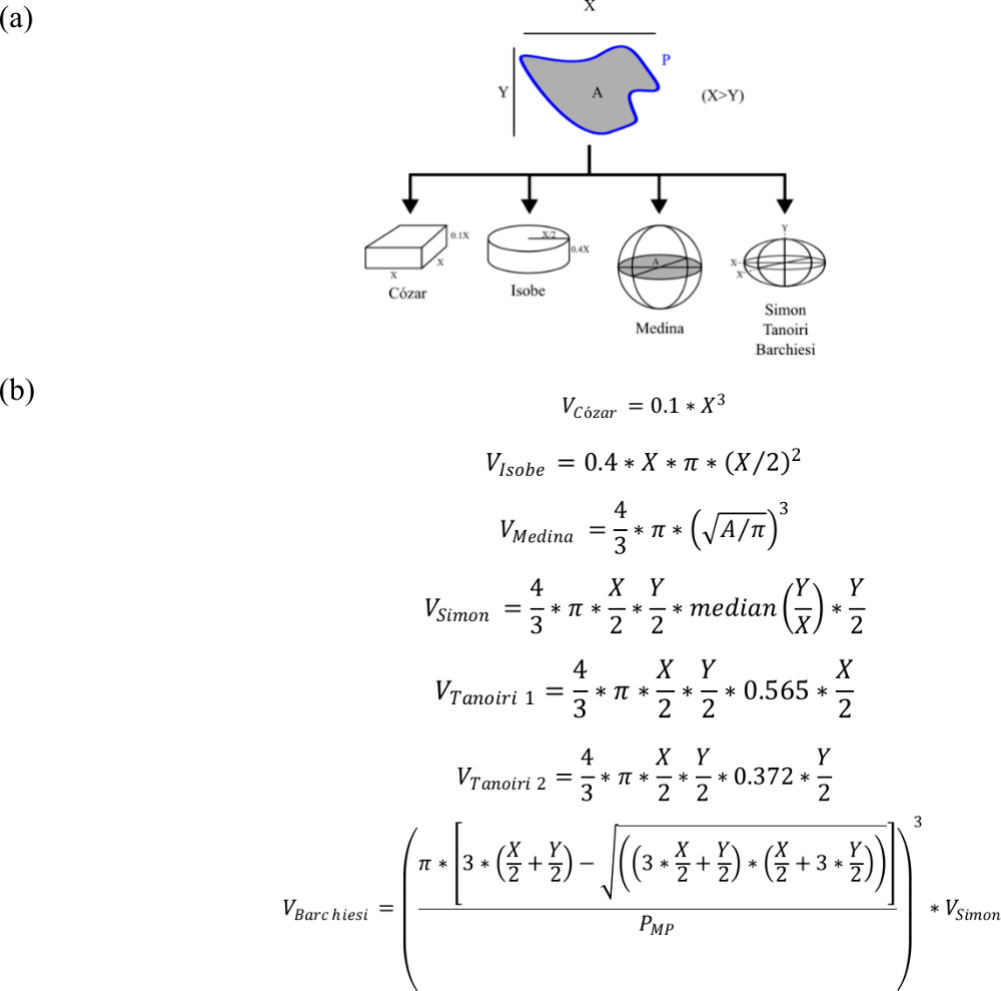
(a) Basic geometrical approaches of the different models considered previously to estimate the volume of MPs from their main 2D projection. (b) Adaptation of the original formulas to estimate the volume (*V*) of a particle using the geometric information provided by the LDIR software (Clarity v. 1.7.17). (Note: *X* is the highest value of height or width, whereas *Y* is the lowest; *A* is the area of the particle, and *P*_MP_ is its perimeter). See text for details.

It is worth noting that defining the “form” of a particle is anything but simple and there have been historically controversies on how to describe it. “Shape” includes every aspect of external morphology (i.e., overall shape, roundness and surface texture).^20^ Further, three intuitive (although complex to unambiguously define) descriptors of particles are “form”, “roundness” and “surface texture”,^21^ which can be differentiated according to the different scales and aspects they consider in the particle.

Form is a first-order property and it reflects variations in the proportions of the particle. Roundness (=smoothness, = roughness) constitutes a second-order property, reflecting variations (presence of) at the corners and edges (which refine the form).^22^ Finally, surface texture (a third order effect, = irregularity)^20^ describes small surface irregularities. Examples of pure shapes are triangles, squares, circles, etc. If the corners of a polygon are rounded, we still have a triangle, square, etc. but with a certain degree of roundness. If the surface is not smooth, but includes small convexities and concavities we have a rough surface (but this does not affect overall roundness nor shape). In principle, shape parameters should be independent of size and take the form of ratios of the axis, which also created problems for their definitions.

In a pioneering study, Cózar et al.^14^ collected environmental samples from 141 sites on the circumnavigating Malaspina expedition in 2010–2011, especially in the oceanic gyres. In total, 3070 plastic fragments (100 cm to 200 μm) were recovered using a 200 μm mesh Neuston net. Raman spectroscopy was used on a particle subset to ascertain their polymeric nature. The size measurements were performed either manually or with the software Zooimage considering only the longest linear lengths. The volume of a collection of particles was estimated approximating them to cubes. Indeed, strictly speaking they considered parallelepipeds as their bases were squared and their heights were fixed at 10% the length of the basis. This statement was based in the morphology of a selected set of particles although not more details were disclosed.

Isobe et al.^16^ attempted to model the global distribution of nonconservative plastics (i.e., plastics that tend to degrade) and how their abundance and distribution change over time. They modeled the particles as cylinders with a height adjusted as 0.4xdiameter of the particle (this constant was selected through trial and error).

Medina et al.^15^ documented the extent and diversity of MPs in aquatic environments and the factors influencing them. They used Raman microspectroscopy and spectral imaging to analyze particles between 1 and 300 μm. The particles were estimated to be spheres with radii equal to half the equivalent diameter of the particles (the equivalent diameter can be defined as the diameter of a circle that has the same area as a given – usually, irregular- particle). They created a 3D map of individual particles; however, this was very time-consuming and it was used only to evaluate the accuracy of the estimations in a very small subset of particles.

Simon et al.^17^ focused on the quantification of the removal rates of microplastics in various Danish wastewater treatment plants using infrared (IR) imaging. They studied particles between 10 and 500 μm and calculated the mass for ellipsoids considering the median of the ratio of the highest and lowest dimensions of the particles. In addition, the height (third dimension) of the ellipsoids was fixed as 67% the minor 2D axis; in addition, cylinders (with 40% void fraction) for the fibers were considered.

Both Tanoiri et al.^18^ and Barchiesi et al.^19^ modified the Simon's model.^17^ The former considered two options depending on whether the particle has an overall ellipsoid or hemiellipsoid form, hereinafter termed Tanoiri1 and Tanoiri2 models, respectively. The model from Barchiesi is a refinement that considers that the surfaces of the particles are irregular, and so should occur for the ellipsoids representing them.

Recently, an approach based on artificial neural networks (ANNs) was employed to decide on the form of the microplastics and to estimate their mass.^23^ However, that application would need a specific training for each environmental application (since the weights of a neural network are sample-dependent) to estimate the thickness (the third dimension of the particles) and to select the best model among several (including those depicted above). Another problem inherent to ANNs is the stochastic initial setting of the weights of the neurons, which complicates the reproducibility of the models when training is repeated several times or when different samples to get a model are considered elsewhere. Further, a quite numerous set of samples would be needed to train the ANN (particularly when spectral data are used) and to validate it. Otherwise overtraining and memorization would occur. This can be a real problem in many practical studies with field samples. Hence, although the results are promising, the routine application of the approach is not straightforward. Also recently, an application called YABE^24^ was developed to process LDIR outputs using the model developed by Barchiesi et al.^19^ and it allows the user introducing a density value for the polymers, if desired. On the other hand, another application called SiMPle^25^ implements the approach defined by Simon et al.^17^

A common limitation for those models (but for that using neural networks), as it can be inferred from the explanations above, is the assumption of a unique geometry for all the particles and – in some cases- a lack of differentiation between fibers and fragments, but for the Simon’s and Barchiesi’s models. The need to estimate the third dimension (height or thickness) is a common major problem that cannot be avoided unless 3D images or dynamic ones can be obtained,^22^ which is not still available for current environmental studies and, so, it remains as an unavoidable problem. Indeed, the third dimension is estimated assuming that the particle under study has a given shape, with a given regularity and with some roundness and surface irregularity. It is also considered that the particle is in a given position (usually, the most stable one, along the two major axes). As long as the particles are isometric (regular) the estimation of the third dimension out of the 2D measured axes constitutes a reasonable approach with <10% error.^22^ Unfortunately, many microplastics do not have regular shapes.

A further controversy, which is beyond the scope of the present work, is the estimation of the density of the polymers. For the purposes of this work we will adhere to the usual consideration that, since polymer degradation is a surface phenomenon, the density of the bulk particle does not change substantially regarding a pristine one. We acknowledge that this is a limitation for all models available nowadays (and likely it will continue to be so because it is not possible to measure the density of each and every microplastic) and it deserves more specific studies.

The objectives of the present work are 4-fold: first, to assess the performance of existing approaches when estimating the mass of a suite of exemplary collections of microplastics, measured by a state-of-the-art tunable quantum-cascade laser-based LDIR system, considering the several 2D geometric parameters it reports. Second, to refine existing models so that they yield more accurate results. Third, to develop a hybrid model by which the shape of each MP is decided upon measuring its geometrical parameters (instead of fixing a general shape from the start). Fourth, to develop an Excel-based application (NOMME, Number Of Microplastics and their Mass Estimation) to classify the MPs identified by the imaging system by polymer type, size range and morphology (fiber/parallelepiped/spheroid) and estimate their mass considering specific models. Noteworthy, routine application of this approach does not require preliminary, sample-dependent, calibration studies.

It is worth noting that other spectroscopic techniques than LDIR instruments can benefit from this application, like hyperspectral cameras or Raman spectroscopy imaging systems. In effect, as long as the polymers have been identified and the necessary geometric parameters were measured (in fact, they are very basic geometrical descriptors), the instrumental outputs can be exported to a CSV (comma-separated values) file or an Excel one, and – if required- the numerical columns reordered to fit the NOMME application.

Furthermore, in this work we contribute to two relevant gaps previous approaches present:

(i) A somewhat lack of model validation when relatively small particles are considered, as Barchiesi et al.^19^ acknowledged. In particular, we will consider particles ranging 20–1500 μm, as opposed to 300–100000 μm,^14^ 200–5000 μm,^16^ 1000–5000 μm^18^ and 500–5000 μm.^19^ Medina et al. considered 1–200 μm, but only 31 particles^15^ and the model could not be validated extensively, and Simon considered 10–500 μm but the model yields overestimations for the highest particles.^19,25^

(ii) The total mass of the polymers considered so far was a bit high and not very similar to the studies performed with chemical imaging (in particular, LDIR systems), where only some micrograms of polymers are usually present over the reflective plates. Therefore, the mass we considered ranged 190–9400 μg, in contrast to values of 0.02–7 g reported by Barchiesi et al.^19^ The other models did not report mass values (except for that from Medina et al., who worked in the nanogram range, as they used Raman spectroscopy). A recent study devoted to evaluate the accuracy of several geometric assumptions of common application employed relatively big micro- and meso-plastics (1–25 mm), at the milligram range,^26^ which are a bit far from the low size range considered in this work.

## Experimental Section

### Samples

The MPs that yielded the exemplary samples were obtained as powders from the eight most broadly used (and environmentally ubiquitous) polymers: HDPE, PA, PC, PET, PMMA, PP, PS, and PVC. They represent more than 70% of the worldwide polymer production^27^ and were also considered by the European Union as of priority concern for their surveillance.^13^

They were obtained in the framework of the European Union-JPI Oceans BASEMAN project^28^ and they contained no additives, but for the indispensable ones to synthesize them. Their main characteristics, as provided by the manufacturers, are resumed next: PS was fabricated by INEOS Styrolution (commercial name: Styrolution PS 158 N/L, 40–350 μm, density: 1.04 g/cm^3^); PP was from Borealis (commercial name: HL508FB, no size distribution specifications, density: 0.91 g/cm^3^); PVC proceeded from Vinnolit Gmbh (commercial name: Vinnolit S3268, 30–300 μm, density: 1.38 g/cm^3^); PET was from Neogroup (commercial name: Neopet 80, 7–40 μm, density: 1.38 g/cm^3^); HDPE was fabricated by LyondellBasell (commercial name: Lupolen 4261AGUV, no size distribution specifications, density: 0.945 g/cm^3^); PA6.6 was from BASF (commercial name: Ultramid, 30–400 μm, density: 1.13 g/cm^3^); PMMA proceeded from Plexiglas (commercial name: Plexiglas 7N, no size distribution specifications, density: 1.19 g/cm^3^); and PC was from Bayer (commercial name: Makrolon 2558, no size distribution specifications, density: 1.20 g/cm^3^).

Recall that we could not work with collections of particles of narrow specific sizes and, so, we could not obtain the true mass corresponding to different specific particle sizes. That study would be an interesting experiment, but we could not perform it. Particles were withdrawn from the containers and poured over the Kevley slide. Then, they were counted and visualized “*ex-post*” using the LDIR system (once they were measured).

Ternary and multiple mixtures of the polymers were prepared to simulate different experimental situations that can appear in real monitoring studies. Several trials were designed: Tests 1 to 9 were performed using only one polymer type, Tests 10 and 11 used ternary mixtures and, finally, Tests 12 to 15 mixed all polymers. The tests and their characteristics are summarized in Table 1.

**Table 1:** Summary of the Microplastic Collections Employed in the Different Trials

Test	Polymer	Weight (μg)	Number of Particles	Diameter Range (μm)
1	PP	9400	934	1500–20
2	HDPE	2240	247	1460–20
3	PS	1480	269	710–20
4	PVC	300	456	360–20
5	PS	800	150	600–20
6	PA66	190	87	550–20
7	PC	1160	201	800–20
8	PMMA	540	174	600–20
9	PET	960	642	660–20
10	HDPE, PS, PET	1720	446	1760–20
11	PC, PA66, PMMA	2020	344	920–20
12	All	5440	1718	1300–20
13	All	2350	789	1500–20
14	All	2650	1280	1200–20
15	All	500	820	650–20

Weighing was performed with a Sartorius ME215P semimicro analytical balance, with a sensitivity of 10 μg (repeatability, 40 μg), in a dedicated cabinet. The weighing was done over a Petri dish containing the dedicated slide support of the instrument plus the Kevley slide itself. After weighing, the sample was introduced in the LDIR system. The Petri dish was weighed before and after taking the sample off, in order to assess possible particle losses during weighing, manipulation and transport to the LDIR instrument. The models presented here will be developed using microplastics collections #1 to 11 and validated with collections #12 to 15. These tests were designed to take into account several variables: different polymers and polymer mixtures, mass, size, number of particles, formation of clusters of particles due to aggregation, etc. However, several factors could not be assessed fully, like different proportions of polymers, biofouled MPs or highly weathered particles. These latter issues might affect the application of our model to complex field matrices. Nevertheless, we think that they affect more the previous identification stage than the calculation of the mass itself (but for the discussion about the true density of the specimens). No doubt, a defective identification of weathered polymeric particles will result in an overall defective estimation of the mass, but that would not be caused by the models considered here.

### Instruments

The measurements were performed using an 8700 tunable quantum-cascade laser-based chemical imaging LDIR system, from Agilent Technologies, in the mid-IR region (1800–975 cm^–1^), with its associated software: Clarity (v. 1.7.17). The particles were deposited over silver-coated reflective MirrIR microscope slides.^29^ The instrumental conditions were the usual ones: “Particle analysis” working mode, wavenumber for the compilation of the preliminary IR image: 1442 cm^–1^, particle size working range: 20–5000 μm, sensitivity: 8, autoscan (no image collection), scan speed: fast, sweep speed: fast. Note that the lowest recommended size threshold for routine measurements in LDIR systems was set at 20 μm, hence the lowest value given in Table 1. There could be a source of error in the mass estimations whenever smaller particles (<20 μm) are present in the Kevley slide because they will not be measured (and so their mass estimated). Nevertheless, the mass proportion of such particles is expected to be very low, as evidenced in some tests whose results are shown in Figure S1, Supporting Information.

For readers not familiar with the LDIR instrument, the “particle analysis” option launches an automatic workflow that, first, detects all the particles above a given size threshold in a selected area; second, measures their geometrical parameters and their spectra, and third, identifies them using spectral libraries. Once the workflow is completed, the user can export the results to an Excel file with the following sheets and corresponding columns:

(i) Sheet 1 contains general information: Analysis Name, Instrument Name, Serial, Software, Version, Analysis Date, Report Date, Particles Analyzed, Wavenumber.
(ii) Sheet 2 contains relevant geometric information: Width (μm), Height (μm), Diameter (μm), Aspect Ratio, Area (μm^2^), Perimeter (μm), Eccentricity, Circularity, Solidity, the Quality index­(=HQI) of the matches; and the polymer identification (column heading = identification), along with additional information not relevant for the calculations (Particle#, Id, Notes, Match Type) (see Figure S2, Supporting Information).

### Geometric Parameters

The main parameters reported by the LDIR to describe the size and morphology of the particles are described briefly next and illustrated in Figure 2:

(i) the height and width corresponding to the main dimensions of the smallest possible rectangle that encloses the particle (note that although the term “height” is used by the software it must be understood as the length of the rectangle);
(ii) the perimeter and area of the particle;
(iii) the diameter, calculated as the equivalent diameter of a circumference with the same area as the original particle (this is different to the Feret’s diameter, which is used in other systems, defined as the distance between two parallel tangential lines to the particle^22^);
(iv) the solidity (ranging 0–1), defined as the ratio of the particle area to its convex hull. Hence, $Solidity=\frac{Area_{\left(Particle\right)}}{Area_{\left(Convex\;Hull\right)}}$ . The convex hull can be visualized as the area that surrounds more closely, and externally, a particle (Figure 2); i.e., without considering the convexities (cavities) of the particle. There are different algorithms to define the convex hull and calculate its area,^30,31^ most of them using the coordinates of the external vertexes. Thus, when solidity is close to unity it can be deduced that the particle has not relevant irregularities on its perimeter (and, so, on its surface). In this sense, solidity can also represent the roughness of the particle as values close to zero denote very irregular particles. More advanced calculations for roughness were proposed elsewhere.^32^ Despite solidity has not been considered often in literature (an exception can be found at^33^) roundness did. However, the usual meaning for roundness corresponds to what nowadays is defined as circularity, not really to the concept of roughness, and this created some confusion in literature;
(v) the circularity (ranging 0–1) describes how far a particle resembles a perfect circle (i.e., how rough it is). It can be calculated as the ratio of the perimeter of a circumference whose area equals the 2D area of the particle to the perimeter of the particle itself; namely, $ularity=4\times \pi \times \frac{Area}{Perimeter^{2}}$ . In fact, this should be called the circularity index or Cox’s index because it is the square of the original definition.^34^ Further, circularity can be considered a proxy for the sphericity of a 3D particle^22^ and it was given an important role to describe the shape of the particles in recent papers;^23,26^

(vi) the eccentricity (ranging 0–1) describes how elongated a particle is, it is calculated from the rectangle that surrounds the particle as $Eccentricity=\sqrt{1-\frac{\min imum\;length^{2}}{\max imum\;length^{2}}}$ ;
(vii) the aspect ratio is defined as the Width/Height quotient (where the “height” in the LDIR software corresponds to the length). It is relevant to differentiate between fibers and particles (fibers have an aspect ratio over 3 or below 0.33 ^35^).

**Figure 2:**
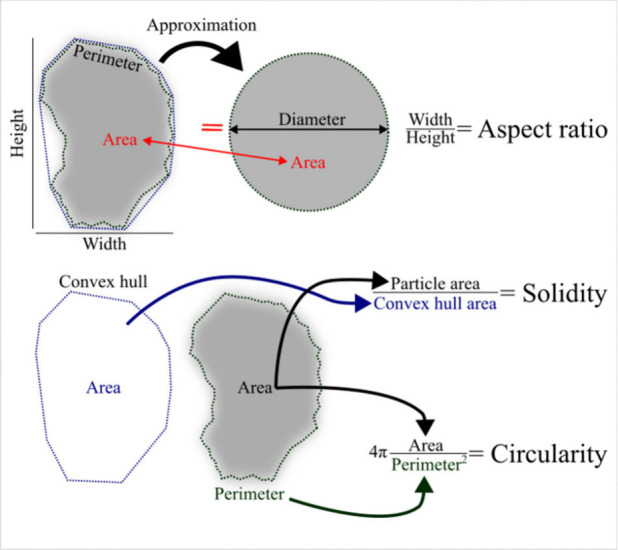
Geometric data reported by the LDIR Clarity Software (v. 1.7.17) relevant for this work.

The volume of each identified polymeric particle was estimated using the models described in the Introductory, whose formulas were resumed previously.^19^ However, they used parameters like “major axis of the best-fit ellipse” and “minor axis of the best-fit ellipse”, so, in order to apply them to the data reported by the LDIR system, they were substituted by the maximum and minimum among the two parameters “height” and “width”, which will be denominated as X and Y respectively. The adapted formulas are shown in Figure 1b.

## Results and Discussion

### Evaluation of the Original Models

When the models reported in literature were applied as such (but for the unavoidable adaptations to use the LDIR outputs, as detailed in the last paragraph of Geometric Parameters), the most straightforward observation was that all of them overestimated the mass of the polymers, even by 2 orders of magnitude (Figure 3).

**Figure 3:**
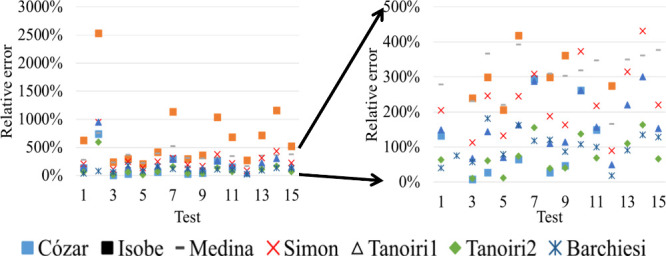
Relative errors (REs, %) encountered for the models tested as they were originally defined (the figure on the right magnifies the region indicated in the left). The numbers in the abscissas correspond to the particle sets indicated in Table 1.

The worst-performing models were those from Isobe and Medina. The former might be optimized by changing the constant (=0.4) in the equation, trying to find out a value which might be best suited to the LDIR measurements. This would require a significant number of trials (which is not practical for routine applications) and, worst, might lead to overfitting the constant to the data sets used here, so we kept the original value. The latter seems to be fundamentally flawed in this case. General consensus^17^ is that virtually all particles in the measurement surface will lie along their largest dimensions, thus the unknown dimension will always be the shortest. This means that the Medina's model, which “converts” the particles into perfect spheres, will always lead to the maximum possible volume. In general, the Tanoiri’s models worked quite well. Their optimization is far from trivial, as pointed out by Barchiesi,^19^ and requires huge workloads that depend on the particular samples at hand, which is not practical for common environmental studies. The Barchiesi’s and Tanoiri2 models yielded the lowest error figures, around 100% overestimations, on average.

Figure 3 shows that many relative errors (RE) surpass 300% (RE= 100·(calculated mass-weighted mass)/weighted mass) and this is coherent with previous evaluations from Primpke et al.^25^ and Tanoiri et al.^18^ who reported up to seven times mass overestimations. Our errors agree very well with a recent paper which reported relative errors between 147 and 830%^23^ for those “traditional” models. Contreras et al.^26^ found 74% and −8% errors for the Simon’s and Barchiesi’s models but remember that they considered large microplastics and high mass (ca. 7 g). Two major reasons that might justify these errors are the inadequacy of the models to estimate the real shape of the particles, and/or the irregularity of the particles that cause the measured lengths to not be totally accurate. The former issue will be addressed in the next section. The latter was already considered by Barchiesi et al. when they corrected Simon’s model with a factor to account for surface irregularity. Indeed, this is the main driving force of their model and it considers the correction factor for the three space dimensions (thus the power of three that can be seen in the equation, Figure 1b).^19^ The problem of surface irregularity is also implicit in the discussion about the many diameters that can be defined for an irregular particle (all being straight-line measurements between parallel tangents on opposite sides (Feret’s diameter), or the distance between opposite sides (Martin's diameter)). Hence, Rosal^22^ recommended considering average lengths derived from the many possibilities. However, this option is highly demanding in both time and workload (mostly when a large number of particles is to be considered, as it is usual in environmental samples).

To improve these results, we implemented several modifications of the models. The first affects only the Cózar model. As seen in Figure 1b, this model considers only the longest dimension (X) of the 2D projection to describe a square and it estimates the third unknown dimension as 0.1*X.^14^ However, as the LDIR system reports both X and Y we decided to calculate the volume of a parallelepiped as V = 0.1·X^2^·Y. This modification improved the results significantly, as the average relative error of the original model was ∼180% whereas with this modification the relative error decreased to ∼58%. Hence, this will be the equation to be applied in this work hereinafter.

On the other hand, the implementation of a “solidity” (as defined in Geometric Parameters) correction factor was considered in the models, as it reflects the irregularities of the 2D projection. Accordingly, it was decided to correct the calculated volumes of the “pure” forms of the models (squares, ellipsoids, spheres, etc.) by multiplying them by the solidity factor. A question arises here as whether solidity should correct two dimensions (width and height, the known 2D dimensions), three (including the estimated height) or just the calculated volume (as defined by the original equations). Therefore, we studied the power that should be considered for the solidity correction. A power of two represents the correction of length and width, a power of three corrects the three main dimensions, whereas a power of four or five are just mathematical extensions of the volumetric corrections.

Figure 4a shows the average relative errors of the different solidity-corrected models. It can be seen that even a mere solidity correction (a power of one, to correct the overall estimated volume) reduces the relative errors of the models. From Figure 4b it is clear that once a power of three is applied, the relative standard deviation – RSD- of the errors does not improve (not even the relative errors themselves – Figure 4a-, but for marginal reductions), and so, a power of three will be considered in the present work. Note that to evaluate the adequacy of a model not only the error of the model was considered, but the relative standard deviation, as well. The reason is that we considered the variability in the mass estimations of the different sets of particles as important as the errors themselves. Thus, we preferred models that led to more consistent errors under different circumstances, which is the reason for the different compositions in the MPs collections (as seen in Table 1). The RSD(%) of the relative errors, was calculated for each studied model as the standard deviation of the relative errors (RE, as defined above) divided by the average relative error. We considered training (n = 11) and validation (n = 4) trials separately.

**Figure 4:**
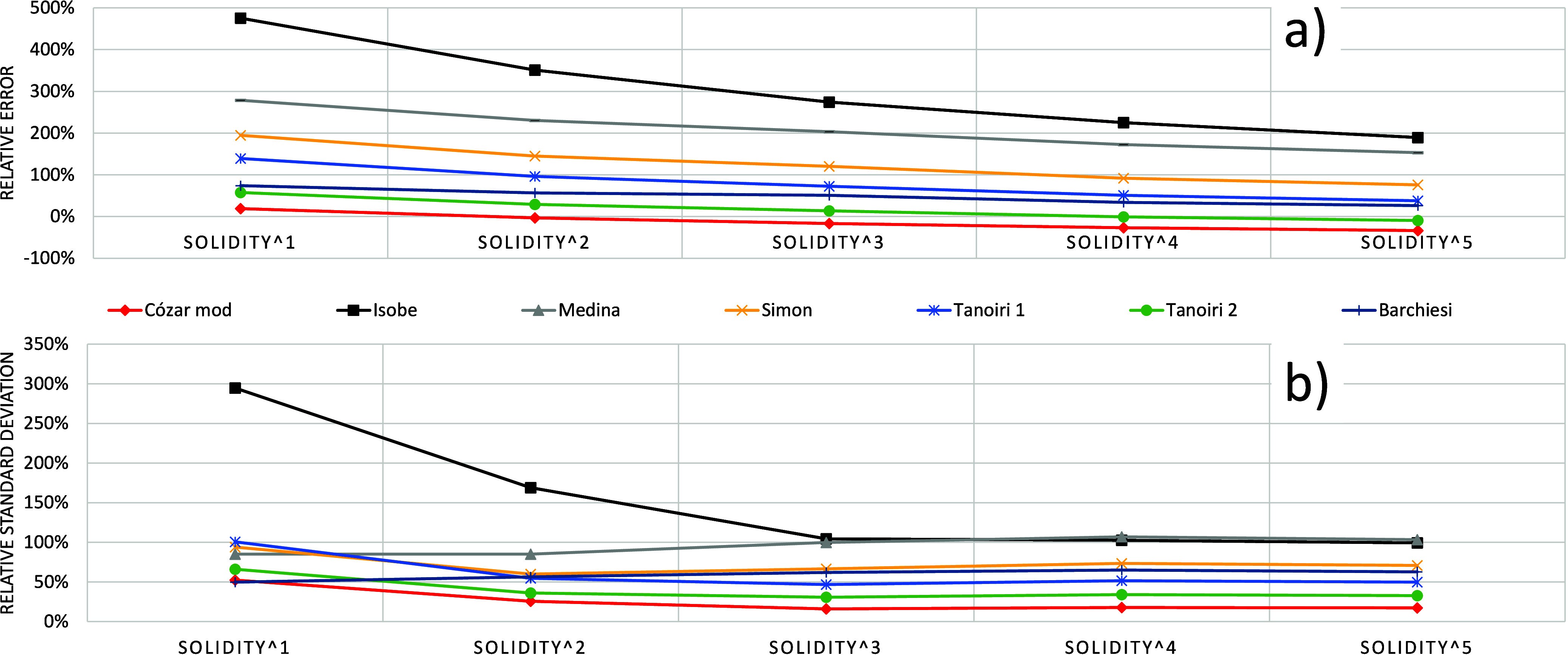
Behavior of the models corrected by different powers of the solidity factor (see text for details). Average relative errors (a) and their relative standard deviation (b) for the calibration trials (*n* = 11).

Figure 5 shows the mass predictions gathered from the tested models when solidity is used to correct each length. In general, relevant reductions of the errors were obtained (compare with Figure 3) and most of them are <100%. As in the previous assays, the worst performing models were Isobe's and Medina's, likely for the same reasons discussed above. The best-performing models were Tanoiri2 (RE = 17%, RSD = 22%) and Cózar – considering the equation as modified above- (RE = 25%, RSD = 35%).

**Figure 5:**
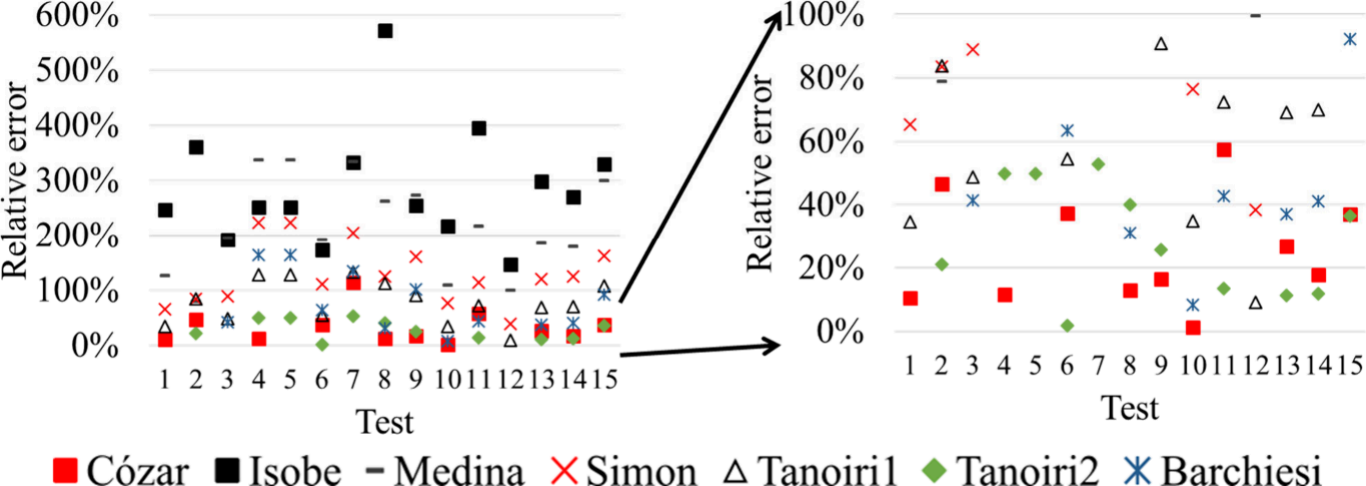
Relative errors of the tested models introducing the correction factor solidity with a power of three (the figure on the right magnifies the region indicated in the left). The numbers in the abscissas correspond to the particle sets indicated in Table 1.

### Hybrid Models

The results obtained above when correcting the geometric factors by the solidity were encouraging but still yielded mass overestimations. A further refinement, which is proposed first time in this work (to the best of the authors’ knowledge), consists of developing a hybrid model. This means that instead of defining a unique volumetric model and, so, establishing a given shape, the volume of each particle is estimated individually, letting its geometric parameters decide on the most appropriate geometric shape that defines it. This idea was also implicit in a recent comment when evaluating the most appropriate shape to describe the microplatics: “carefully considering the shape of each individual particle”;^26^ and in the approach using artificial neural networks.^23^ Hence, different models were applied here to estimate the volume of different MPs. The models selected to perform such an hybridization were Cózar’s and Tanoiri2, for two main reasons: they were, generally, the best-performing models and they offer complementary approximations to different shapes. For fibers, a specific model was developed.

The hybrid model starts by discriminating between fibers and fragments. For this we used the tiered strategy detailed elsewhere,^35,36^ which had been applied also in YABE.^24^ In essence, this corresponds to a primary elucidation of the main shape or overall morphology of the particles (1st level descriptor, as commented in the introduction): fibers or fragments. The MP is considered a fiber when their solidity is lower than 0.35 or if its aspect ratio is over 3 or under 0.33.^35^

To calculate their volume, fibers were considered as cylinders. However, instead of applying the Simon’s calculations (for which we obtained poor results) a sort of “equivalent cylinder” is deduced from an equivalent rectangle (in the same way as the equivalent diameter is defined). As the LDIR software provides the area and perimeter of the fiber, it is possible to use them to calculate the two dimensions of a rectangle whose perimeter and area equal those from the fiber, but without its bends and curls (hence, the term equivalent rectangle). Considering the width as the 2D projection of the diameter of the circular basis the volume of the “equivalent cylinder” is calculated from a second order equation obtained considering perimeter and area. To validate this procedure, four separate trials were performed using fibers from PP ropes of different lengths and widths. Their average relative errors were <64%, which compared favorably with ca. 720% error when using the Simon’s model. This represented a significant improvement but ongoing efforts are done in our laboratory to refine these predictions. Some exemplary comparisons between these models obtained in some trials can be found in the Supporting Information.

It is worth noting here that there are several important sources of error for models dealing with fibers. If the fiber curls over itself, or if several fibers overlap over each other – as it happens frequently-, the image recognition software will not be able to recognize the individual fibers (nor the intermediate void spaces) and, as a result, they will be detected as a “solid” fragment instead, and their volume will be overestimated (see Figure 6). Further, fibers may be partially out of focus, as they tend to not be fully flat and are not always in good contact with the reflecting support. The imaging software may interpret the detected item as a fiber wider than it actually is or it may “cut” the fiber into separated sections – as it happens often- which are counted as different items. Therefore, it is acknowledged that samples with high quantities of fibers may lead to defective mass estimations (not only due to the mass estimation model, but because of a lack of correct previous identification).

**Figure 6:**
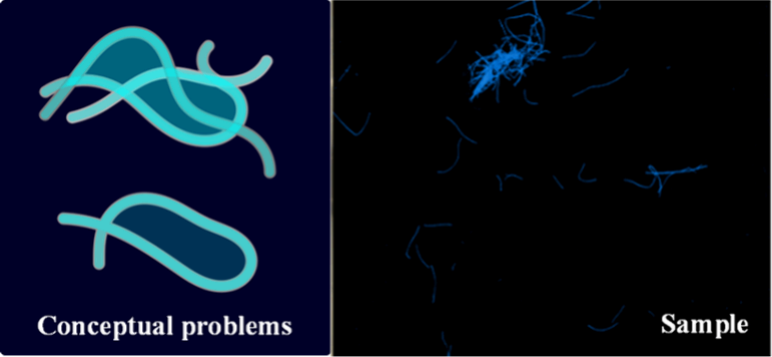
Examples of nonflat and overlapped fibers; common problems encountered when identifying fibers in real samples (conceptual idea and a sample).

The second stage of the hybrid approach consists of assigning a 2D main shape to each fragment. The working hypothesis is that the Cózar model will perform better for parallelepiped-shaped particles (square/rectangular bases), whereas the Tanoiri2 (ellipsoid approximation) will perform better for the rounded particles. This discrimination was performed using the circularity parameter, as detailed in the decision flow presented in Figure 7. The threshold value for the circularity was decided considering different criteria:

(1) Experiments with our collections of particles (Figure 8). There it can be seen that the errors of the predictions get much lower after a circularity of 0.7 is considered, not only for the modeling stage but for its validation.
(2) Careful study of the seminal examples from Cox^34^ (although he discussed about “roundness”, it is considered as the circularity nowadays) where circularity values lower than 0.85 (on average) denote not too circular shapes. Incidentally, note that according to definition, the circularity of a perfect square is 0.785, so this threshold seems reasonable. Further, we also took into account the classical Krumbein’s chart that is used widely in related literature; see, e.g. references.^21,37^

(3) Some reports where the circularity and/or sphericity were studied. In particular, in^38^ it is seen that only high circularity values (>0.9) are closely related to the spheroidal shape (in combination with a high sphericity). In other studies, the spherical shape was restricted only for high values of the different studied parameters.^32,39^

**Figure 7:**
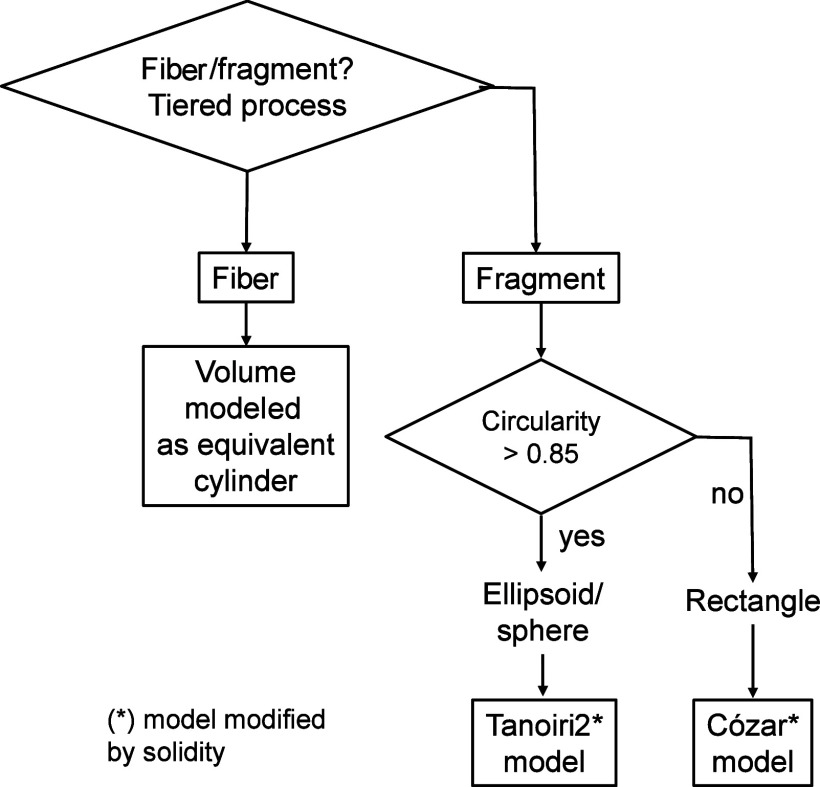
Overall decision workflow to generate the hybrid models using the LDIR outputs. The asterisk means that the models given in refs 8 and 12 were corrected by the solidity in all cases. The differentiation between fibers and fragments follows a tiered approach.^26,27^

**Figure 8:**
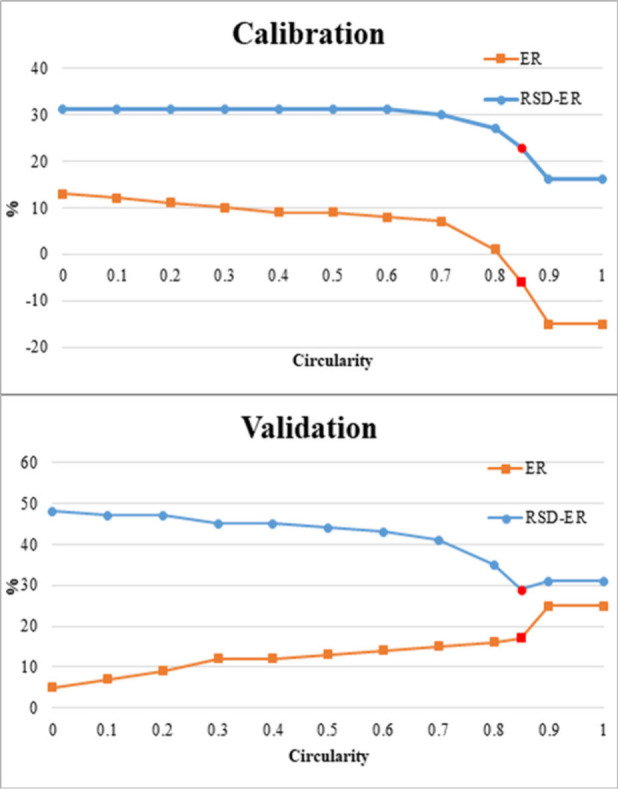
Testing accuracy when estimating the mass of microplastics using the hybrid model and different circularity thresholds. ER = relative error (%); RSD = relative standard deviation (%).

Despite at present many efforts are done to refine those thresholds (see the previous references as examples) they still serve as benchmarks and, so, we considered them.

Note that solidity (and/or its equivalent term, roundness) takes into account the third level descriptors of the particles, as explained above. This obviously impacts the total volume of a particle and, therefore, we considered it to correct the estimated volume of the particles as in previous section.

When this hybrid approach was applied to study the 15 known collections of particles it yielded the following results: for calibration (i.e., the collections of particles used to adjust the models, sets #1 to 11, Table 1), average relative error= −6%, RSD= 23%. For validation (application of the model to totally new sets of particles, sets #12–15), average relative error= 17%, RSD= 29% (see Figure 8). These results clearly outperform those of the individual, original models.

The overall approach to apply the hybrid model was implemented in an Excel-based application called NOMME (Number Of Microplastics and their Mass Estimation, which classifies the MPs identified by the LDIR system and evaluates their mass) and these results suggest that it is a promising approach to estimate the mass of microplastics.

Finally, in an attempt to foresee the reasons why the hybrid approach outstands, the distribution of some geometric descriptors for the test sets was visualized using traditional Box-Whisker plots. Figure 9 exemplifies the results for test set #14. The aspect ratio descriptor indicates that most particles had a ratio around one, which suggested regular fragments (circular or squared) although some of them are clearly fibers (aspect ratio ≫3). The circularity index points out that despite many particles are rounded most are not (circularity <0.75), and that some are not circular at all. Finally, solidity shows that in our example most of the items had not too large superficial irregularities but some do. Therefore, the results suggest that whenever a variety of particles exist in a sample, which is likely to happen in field samples, the hybrid model constitutes a rational approach to model the volume of the identified microplastics. Likely, this workflow is better than assuming a general shape and considering a unique model.

**Figure 9:**
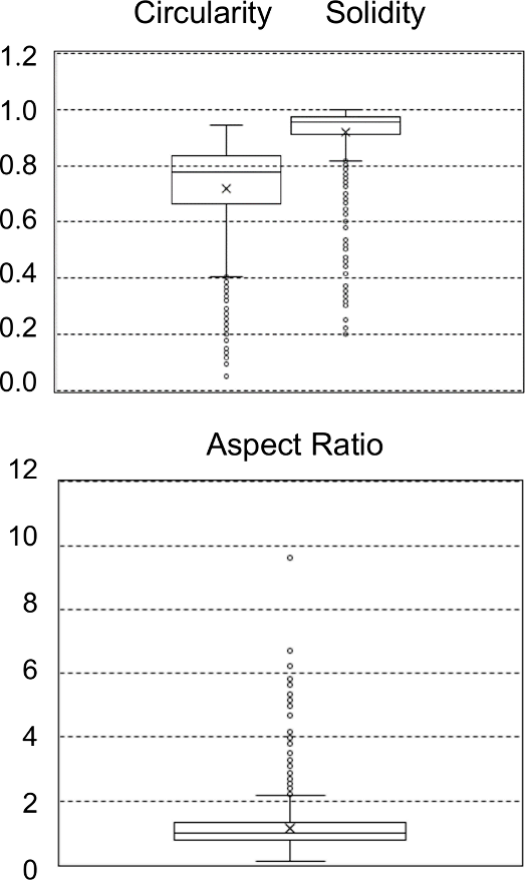
Distribution of some relevant geometric parameters of the particles for test 14 of Table 1.

## Conclusions

Several published mass estimation models were evaluated using 15 collections of particles whose mass were known. They represented different approximations to calculate the volume of the particles (cubes, cylinders, spheres and spheroids) but yielded unsatisfactory results, with high overestimations of the mass of microplastics.

To improve these results several modifications were implemented. First, a change in the model that considers parallelepipeds to take account of both dimensions of their rectangular basis (Cozar’s model). This improved the performance of this specific model significantly (from 179% to 58% relative error). Next, for all models, the estimated volume was multiplied by the solidity factor (to the power of three) to take account of the irregularities of the particles surface. Even though the results improved again, there still was a bias toward mass overestimation.

Finally, a hybrid model that does not require preliminary assays for its routine application was developed. It first discriminates between fragments and fibers, using the solidity and aspect ratio parameters. Then, to estimate the volume of each fiber an approach was developed, the equivalent cylinder, by which a rectangle (equivalent rectangle) is defined so that it has the same area and perimeter than the fiber. The diameter of the cylinder will coincide with the width of the equivalent rectangle. For the fragments, the circularity parameter was used to ascertain their main shape. Studying previous publications, and performing our own trials a threshold of 0.85 was set. Particles with circularity >0.85 are considered spheroids (ellipsoids or spheres) and the spheroid model will be applied, otherwise they will be considered parallelepipeds and their specific model will be used. With this approach the shape of each particle is not decided *ex ante* and the estimation of the mass of microplastics reached average errors below 20%, which is a remarkable result.

A limitation of all the models is the evaluation of the mass (and volume) of fibers. If they are short and dispersed, without significant entanglements, superposition or loss of focus, the imaging system can identify them individually. Then the models can yield satisfactory results. However, if they are long and blurry the imaging system can either detect them as wider than they actually are, do not detect them at all or divide them in multiple sections. Entangled or overlapped fibers, will be identified as “current” particles, producing large overestimations on their volume and mass. Thus, it is recommended to exercise caution when applying mass-estimation models to samples with high concentrations of fibers.

There are future avenues of improvement: an increased number of assays would help fine-tune the constants implicit in the parallelepiped (Cózar’s), cylindrical (Isobe’s) and ellipsoidal (Tanoiri’s) models. However, one must be aware that there is also a risk for overfitting the working examples and getting unreliable models. Also, the models presented here were evaluated with unweathered and not-biofouled particles. Those effects may affect the identification of putative microplastics in field samples and, so, be detrimental for the mass estimation. Nevertheless, note that the main cause of the problem is not the mass estimation models themselves but the difficult identifications before they are used.

The approach described in this paper has been programmed in a compiled Excel-based application: NOMME (Number Of Microplastics and Mass Estimation). It does not require special knowledge from the users – but some basic practice with this spreadsheet- and includes the basic instructions. It uses the output of the Particle Analysis workflow of the LDIR Clarity software to classify the identified particles by polymer, size range, fiber or fragment and to estimate the mass of the microplastics using the hybrid model described in this work. This application offers a reliable and streamlined solution for the reporting of analysis results, following the directives of the EU (polymer type and classification sizes). It also yields the overall mass estimation of the identified microplastics, as described in this work. Next developments will disaggregate mass by polymer and sizes. Non-LDIR users can also apply the program as far as they get geometric parameters from their imaging systems (Height (μm), Width (μm), Area (μm^2^), Perimeter (μm), Circularity, Solidity and Aspect ratio), maybe after some reorganization of the output columns of the spreadsheet. More detailed instructions are shown in the Excel file. NOMME is available for free at Zenodo (10.5281/zenodo.15676697) and at the LabPlas Web site (WP3: Advanced Analytics: https://labplas.eu/about/deliverables/).

## Supplementary Material



## Notes

Exemplary predictions for the mass of fibers; Overall estimated mass predicted for the different trials using the tested algorithms; Two examples showing the distribution of mass as a function of the equivalent diameters of the identified particles; Spreadsheet data format to apply the calculations set in NOMME (PDF)
