# Supplementary material for: Microplastic Mass Estimation Using Two-Dimensional Chemical Images from Quantum-Cascade Laser-Based Infrared Spectrometers
Source: Anal Chem. 2025 Oct 28;97(44):24458–67. doi: 10.1021/acs.analchem.5c04003 (PMC12613143; doi:10.1021/acs.analchem.5c04003)
Supplement: Supplementary file 1 [file ac5c04003_si_001.pdf]

# SUPPORTING INFORMATION

# MICROPLASTIC MASS ESTIMATION USING TWO-DIMENSIONAL CHEMICAL IMAGES FROM QUANTUM-CASCADE LASER-BASED INFRARED SPECTROMETERS

Borja Ferreiro, José M. Andrade (*), Adrián López-Rosales, Soledad Muniategui-Lorenzo

Grupo Química Analítica Aplicada (QANAP), Instituto Universitario de Medio Ambiente (IUMA), Centro de Innovación Tecnolóxica en Edificación e Enxeñería Civil (CITEEC), Universidade da Coruña, Campus da Zapateira, E-15071, A Coruña, Spain

(*) Corresponding author, andrade@udc.es, fax: +34981167065

**CONTENTS:**

1- How the equivalent-cylinder model outperforms previous approaches to estimate the mass of fibres (four examples). S1
2- Overall predictions of the total mass of microplastics in the trials using the tested models. S2
3- Figure SI1: Two examples showing the distribution of mass as a function of different equivalent diameters of the identified particles. S3
4- Figure SI2: The spreadsheet organization of the data columns needed to apply NOMME. S4

## 1. EXEMPLARY PREDICTIONS FOR THE MASS OF FIBRES

In total 4 tests are presented here, all of them using fibres from a PP rope. Three models were tested: the Cózar's model (see Ref number 14 in the main text), the Simon's model (see Ref number 17 in the main text), and the equivalent-cylinder model described in the main article.

Test 1: 15 small fibres, 2-3 mm length. Several out-of-focus sections were observed.

- Measured mass: 810 µg
- Estimated mass (Cózar's model): 3522 µg → 335 % Error
- Estimated mass (Simon's model): $6.4*10^9$ µg → $7.84*10^8$ % Error
- Estimated mass (Equivalent cylinder model): 1576 µg → 94 % Error

Test 2: Thin fibres ~3 mm lenght. Significant entanglements were observed.

- Measured mass: 550 µg
- Estimated mass (Cózar's model): 14035 µg → 2452 % Error
- Estimated mass (Simon's model): $2.51*10^{11}$ → $4.6*10^8$ % Error

- Estimated mass (Equivalent cylinder model): 1321 → 140 % Error

Test 3: 4 long, ~0.4 mm wide fibres. No significant out-of-focus sections nor entanglements.

- Measured mass: 6210 µg
- Estimated mass (Cózar's model): 2992 µg → 52 **%** Error
- Estimated mass (Simon's model): $7.95*10^{10}$ µg → $1.3*10^{7}$ **%** Error
- Estimated mass (Equivalent cylinder model): 6883 µg → 11 **%** Error

Test 4: 3 long, ~0.4 mm wide fibres. No significant out-of-focus sections nor entanglements.

- Measured mass: 2130+2390+2400= 6920 µg
- Estimated mass (Cózar's model): 3822 µg → 45 **%** Error
- Estimated mass (Simon's model): $7.95*10^{10}$ µg → $1.3*10^{7}$ **%** Error
- Estimated mass (Equivalent cylinder model): 7705 µg → 11 **%** Error

## 2. OVERALL ESTIMATED MASS PREDICTED FOR THE DIFFERENT TRIALS USING THE TESTED ALGORITHMS

When two values are presented in a cell, the upper one corresponds to the original model whereas the second corresponds to that model when the solidity correction described into the main text was applied.

| | Measured weight (µg) | Cózar | Mod. Cózar | Isobe | Medina | Simon | Tanoiri1 | Tanoiri2 | Barchiesi | Hybrid |
|---|---|---|---|---|---|---|---|---|---|---|
| Test 1 PP | 9400 | 21831<br>10383 | 12661<br>6431 | 68585<br>32619 | 35600<br>21342 | 28714<br>15548 | 15388<br>8332 | 23371<br>12655 | 13268<br>8694 | -<br>6518 |
| Test 2 HDPE | 2240 | 18755<br>3286 | 11933<br>2067 | 58920<br>10323 | 19125<br>4006 | 23516<br>4111 | 15508<br>2711 | 23553<br>4117 | 3929<br>907 | -<br>2068 |
| Test 3 PS | 1480 | 1602<br>1378 | 1139<br>997 | 5032<br>1683 | 4904<br>1689 | 3148<br>1095 | 1633<br>568 | 2480<br>862 | 2326<br>816 | -<br>1067 |
| Test 4 PVC | 300 | 381<br>335 | 300<br>275 | 1197<br>1052 | 1401<br>1312 | 1040<br>969 | 483<br>450 | 733<br>683 | 846<br>792 | -<br>431 |
| Test 5 PS | 800 | 780<br>694 | 585<br>527 | 2450<br>2182 | 2566<br>2337 | 1862<br>1692 | 895<br>814 | 1360<br>1236 | 1430<br>1307 | -<br>635 |
| Test 6 PA66 | 190 | 313<br>261 | 225<br>193 | 984<br>821 | 935<br>826 | 656<br>577 | 303<br>290 | 501<br>441 | 498<br>444 | -<br>216 |
| Test 7 PC | 1160 | 4573<br>2478 | 2538<br>1390 | 14368<br>7786 | 7219<br>4196 | 4742<br>2601 | 2961<br>1624 | 4497<br>2466 | 2528<br>1522 | -<br>1391 |
| Test 8 PMMA | 540 | 687<br>610 | 502<br>451 | 2157<br>1916 | 2214<br>2013 | 1554<br>1409 | 748<br>678 | 1137<br>1030 | 1192<br>1087 | -<br>527 |
| Test 9 PET | 960 | 1408<br>1118 | 962<br>790 | 4424<br>3511 | 3876<br>3315 | 2523<br>2116 | 1354<br>1135 | 2057<br>1725 | 1801<br>1556 | -<br>866 |
| Test 10 Mix | 1720 | 6222<br>1740 | 3543<br>1137 | 19546<br>5465 | 7204<br>3596 | 8129<br>3035 | 4094<br>1529 | 6218<br>2322 | 3563<br>1863 | -<br>1176 |
| Test 11 Mix | 2020 | 5038<br>3183 | 2860<br>1859 | 15827<br>10000 | 9035<br>6395 | 6421<br>4312 | 3415<br>2294 | 5187<br>3484 | 4041<br>2886 | -<br>1885 |
| Test 12 All | 5440 | 6499<br>4286 | 4043<br>2842 | 20418<br>13466 | 14445<br>10848 | 10293<br>7529 | 5350<br>3913 | 8125<br>5943 | 6424<br>5000 | -<br>6518 |
| Test 13 All | 2350 | 6128<br>2978 | 3798<br>1928 | 19253<br>9356 | 10584<br>6725 | 9759<br>5159 | 4948<br>2615 | 7514<br>3972 | 4487<br>3222 | -<br>2068 |
| Test 14 All | 2650 | 10641<br>3123 | 5755<br>2105 | 33429<br>9812 | 12225<br>7426 | 14085<br>5976 | 6986<br>2964 | 10611<br>4502 | 6214<br>3737 | -<br>1067 |
| Test 15 All | 500 | 989<br>685 | 618<br>477 | 3106<br>2150 | 2388<br>1996 | 1602<br>1313 | 833<br>683 | 1265<br>1037 | 1140<br>961 | -.<br>431 |

## 3. Figure S1: TWO EXAMPLES SHOWING THE DISTRUBUTION OF MASS AS A FUNCTION OF THE EQUIVALENT DIAMETERS OF THE IDENTIFIED PARTICLES

Notes: (i) “Test” refers to the experimental trial (as numbered in Table 1 of the paper);

(ii) The estimated mass and number of identified particles for each diameter size range were normalized by the total estimated mass and the total number of identified particles, respectively.

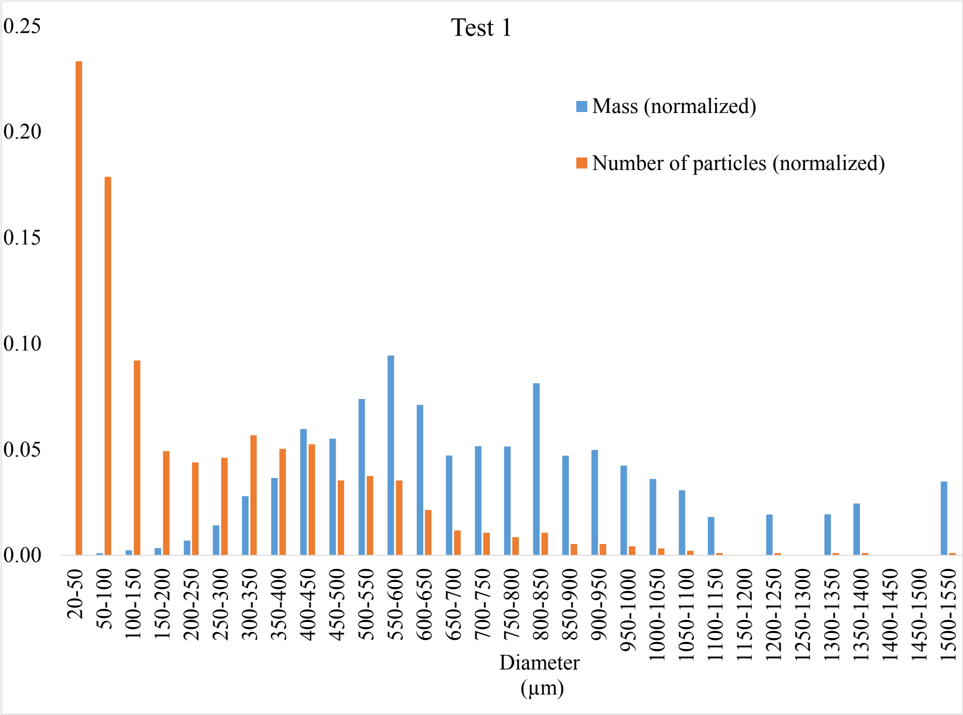


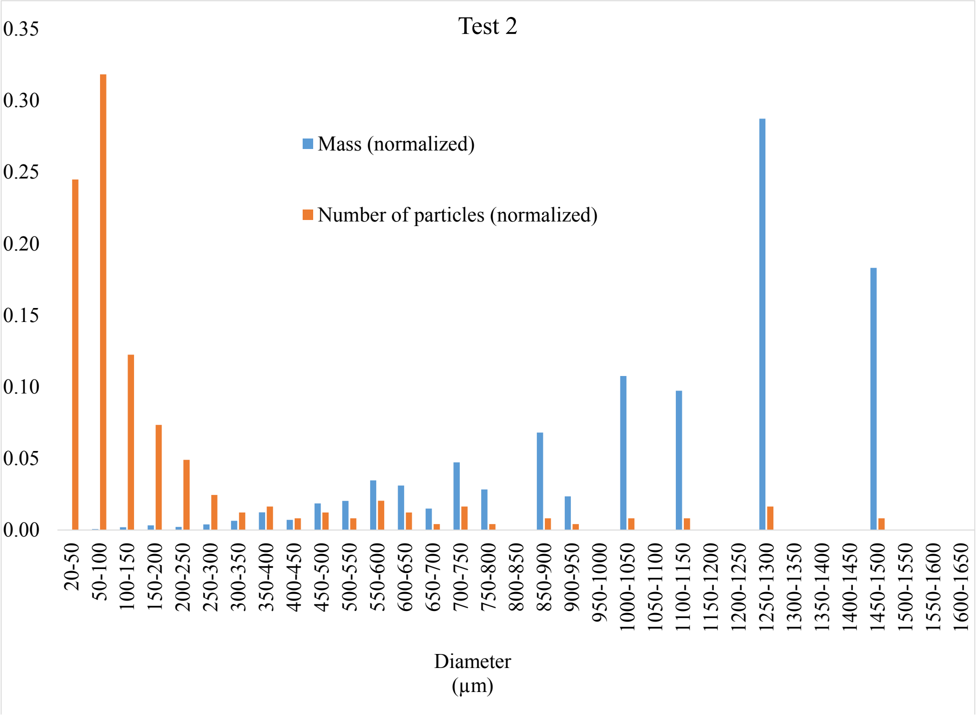

## 4. Figure S2: SPREADSHEET DATA ORGANIZATION TO APPLY THE CALCULATIONS SET IN NOMME

| | A | B | C | D | E | F | G | H | I | J | K | L | M | N | O | P |
|---|---|---|---|---|---|---|---|---|---|---|---|---|---|---|---|---|
| 1 | # | Id | Width (µm) | Height (µm) | Diameter (µı | Aspect Ratio | Area (µm²) | Perimeter (µ | Eccentricity | Circularity | Solidity | Identificatio | Notes | Match Type | Quality | Is Valid |
| 2 | 1 | A1 | 3386 | 19226 | 2616.27065 | 0.17613108 | 5375950 | 49153.5689 | 0.96877119 | 0.02796115 | 0.12948823 | PP | | Auto | 0.8073069 | true |
| 3 | 2 | A2 | 545 | 95 | 174.397562 | 5.7148846 | 23887.5 | 2037.31492 | 0.83542403 | 0.07232097 | 0.54074703 | PP | | Auto | 0.78724228 | true |
| 4 | 3 | A3 | 45 | 75 | 58.3602243 | 0.6 | 2675 | 210.710677 | 0.69595459 | 0.75711276 | 0.94690265 | Cellulose (Ca | | Auto | 0.69418349 | true |
| 5 | 4 | A4 | 110 | 40 | 48.5334231 | 2.77165359 | 1850 | 342.132031 | 0.88913092 | 0.1986068 | 0.59919028 | PP | | Auto | 0.82918582 | true |
| 6 | 5 | A5 | 33 | 95 | 48.5334231 | 0.34494772 | 1850 | 230.710677 | 0.8097042 | 0.43676337 | 0.79569892 | PU foam | | Auto | 0.70663329 | true |

Column A: # (Not relevant) → void or any alphanumeric string

Column B: Id (Not relevant) → void or any alphanumeric string

Column C: Width (µm)

Column D: Height (µm)

Column E: Diameter (µm)

Column F: Aspect ratio

Column G: Area (µm$^2$)

Column H: Perimeter (µm)

Column I: Eccentricity

Column J: Circularity

Column K: Solidity

Column L: Identification

Column M: Notes (Not relevant) → void or any alphanumeric string

Column N: Match type (Not relevant) → void or any alphanumeric string

Column O: Quality (this is the Hit Quality Index)

Column P: Is Valid (Not relevant) → void or any alphanumeric string
